# Supplementary material for: Bifurcation and optimal control analysis of HIV/AIDS and COVID-19 co-infection model with numerical simulation
Source: PLoS One. 2023 May 5;18(5):e0284759. doi: 10.1371/journal.pone.0284759 (PMC10162571; doi:10.1371/journal.pone.0284759)
Supplement: Attachment — Submitted filename: PLOS COVID^MHIV.pdf [file pone.0284759.s002.pdf]

Review Report

Title: Bifurcation Analysis and Optimal Control for COVID-19 and HIV/AIDS Co-Infection

The authors developed a mathematical model with 11 non-compatible compartments to study the transmission dynamics of both COVID-19 and HIV/AIDS. This compartmental model is a significant contribution to the field as it provides a more detailed understanding of the transmission dynamics of COVID-19 and HIV/AIDS in coexistence. The authors' use of 11 non-compatible classes enhances the accuracy and depth of the analysis, offering new insights into the complex relationships between these two diseases.

My comments for the manuscript are below:

Abstract

1. The author could not present the main finding of the study. The failure to present the study's key results detracts from the overall impact and relevance of the research. To make the study more impactful, the author should emphasize the main findings and highlight their significance in the context of the field.

Introduction

2. The manuscript lacks sufficient detail regarding co-infection. Information regarding the effects of simultaneous infection with both COVID-19 and HIV/AIDS, the body's physiological responses, and the underlying chemical processes is missing.

3. In the second from last paragraph

   The first sentence starts with "Theoretically, they looked at the single infections infection-free equilibrium points' local asymptotically stability if their estimated basic reproduction numbers were less than unity." It seems to be not clear; what do you mean by this sentence

4. The last paragraph needs more evidence and how sure are that "Despite the fact that many researchers devote significant time and effort to researching HIV/AIDS, none of them has considered COVID-19 protection, COVID-19 vaccination, COVID-19 quarantine with treatment, HIV protection, and HIV treatment as prevention and control strategies in their model formulation. Furthermore, no other academics have conducted this research, and the above-mentioned literature has encouraged and empowered us to do this research and pick up the slack"
5. The organization of the work may not be deemed necessary and can be removed.

Section 2: mathematical model construction

6. Check the condition for modification parameter $\omega_2 > \omega_1 > 1$ and what are their numerical values, seem to miss from Table 3.
7. In Figure 1: The schematic diagram of the COVID-19 and HIV Co-infection transmission
   - It's not clear where do C_q come from (susceptible or infected class)
   - which mechanism is used to transfer individuals from class H_u to H_a at the rate of theta?. Are there any screening issues? Could you explain, please
   - How do individuals move from class S, H_p and C_v to class C_i how this possible? Is there any mechanism such as screening, testing etc used to validate this?
   - The class C_t is mentioned but does not appear in the model compartment
   - Class R is not defined

   This model needs to be reworked
8. Analytical Result of the Models: This section requires revision to align with the model's formulation.

9. Analysis of the Optimal Control Strategy: The manuscript appears to be lacking the following items, please consider incorporating them
   - Existence of an optimal control
   - Uniqueness of the Optimal Control Solution

10. In Table 3. The numerical value for is 0.2 not 0,2
11. The figure captions lack adequate information and consistency in the heading of the figures
12. Writing language (typos, grammar etc.) and editing need to be seriously reviewed.

Decision: Given these concerns, a comprehensive revision of the manuscript is highly recommended.
