# Supplementary material for: Bifurcation and optimal control analysis of HIV/AIDS and COVID-19 co-infection model with numerical simulation
Source: PLoS One. 2023 May 5;18(5):e0284759. doi: 10.1371/journal.pone.0284759 (PMC10162571; doi:10.1371/journal.pone.0284759)
Supplement: Attachment — Submitted filename: Report.docx [file pone.0284759.s001.docx]

**Report**

**Reviewer**

- **After checking the paper, I found it suitable for publication in this journal with some modifications.**

1. Abstract of the article should contain method, result and conclusion. In this article, there is no concept of optimal control theory. Please add about it.
2. In introduction part review some papers regarding optimal control of COVID-19, HIV and their confections using one paragraph since you are talking about optimal control as well in the paper.
3. In equation (8), you concluded the LAS of DFE before express b interims of basic reproduction number. Therefore, express b interims of basic reproduction number before your conclusion.
4. I know that GAS of EEP is somewhat difficult; however, you should add GAS of DFE of co-infections.
5. In equation (14), what is the difference between forward and back-ward bifurcation? I need detail explanation.
6. In optimal control theory, what does mean the set of admissible control functions? Why control variables are Lebesgue measurable? Why not Riemann measure? I need explanations.
7. In theorem (8), the proof is not clear. Try to separate the proof and explain using Pontryagin minimum principle.
8. In conclusion, there is no result which is connected with the concept of optimal control theory. Please add about it.
9. What about the existence of optimal controls? State the theorem and leave the proof.
10. In numerical simulation of optimal control part: describe the concept forward-backward fourth order Runge Kutta method. I hope you used this numerical method. Moreover, in the objective functional of your problem, you minimized 4 populations, but your graph is only one compartment. Simulate all graphs side by side. See the articles mentioned below.
11. Some work that can use for this literature especially on optimal control theory.

- T. D. Keno, O. D. Makinde and L. L. Obsu, Optimal Control and Cost Effectiveness Analysis of SIRS Malaria Disease Model with Temperature Variability Factor, *Journal of Mathematical and Fundamental Sciences,***53**(1), 134-164, 2021.
- T. D. Keno, L. B. Dano, and O. D. Makinde. Modeling and Optimal Control Analysis for Malaria Transmission with Role of Climate Variability, *Computaional and Mathematical Methods*, 2022.
- T. D. Keno, O. D. Makinde and L. L. Obsu, Modelling and Optimal Control Analysis of Malaria Epidemic in the Presence of Temperature Variability, *Asian-Europian Journal of Mathematics*, **15**(1), 2022.
