# Supplementary material for: Bifurcation and optimal control analysis of HIV/AIDS and COVID-19 co-infection model with numerical simulation
Source: PLoS One. 2023 May 5;18(5):e0284759. doi: 10.1371/journal.pone.0284759 (PMC10162571; doi:10.1371/journal.pone.0284759)
Supplement: Attachment — Submitted filename: Response For reviwiers.docx [file pone.0284759.s004.docx]

***Reviewer comments and our reflections for the comments***

First of all we would like to say thank you so much for taking the time to review our manuscript and provide us with your valuable feedback. We genuinely appreciate your thoughtful contributions and values the effort you have put into making this project a success. Your feedback and insight have been invaluable, and we are truly grateful for your support and input. Thank you for believing in us and helping us reach our goals. We have stated the requested correction and our reflection as follow.

**Reviewer #3:**

1. Theorem 6 states existence of the backward bifurcation but didn’t you simulate that process to show it visually?

**Reflection**: As per the request of the reviewer comment in the revised manuscript we have demonstrated the existence of back ward bifurcation using simulation.

2. There are some typos in the text starting with Abstract with in the 3rd line: “is formulated and analyzed analyzed. “and the paper needs a professional language editor to correct it in an improved english language.

**Reflection:** We have edited to correct our mistakes in the whole manuscript accordingly.

3. The main research topic in the manuscript together with general approach adopted and used by the authors deserve to be given credit.

**Reflection:** We are strongly believe that all the authors guide us deserve credit so that, in the revised manuscript we have incorporated all the concepts mentioned.
